# Risk of Chronic Kidney Disease and Implications in Patients with Atrial Fibrillation for the Development of Major Adverse Cardiovascular Events with Machine Learning

**DOI:** 10.3390/medsci13040289

**Published:** 2025-11-27

**Authors:** Pedro Moltó-Balado, Josep-Lluís Clua-Espuny, Carlos Tarongi-Vidal, Paula Barrios-Carmona, Victor Alonso-Barberán, Maria-Teresa Balado-Albiol, Andrea Simeó-Monzó, Jorge Canela-Royo, Alba del Barrio-González

**Affiliations:** 1 CSI Llíria, Departament de Salut de Arnau de Vilanova, Conselleria de Sanitat, 46160 Valencia, Spain; albadelbarrio17@gmail.com; 2 Clínica Equilibria, 46160 Valencia, Spain; 3 Ebrictus Research Group, Research Support Unit Terres de l’Ebre, Institut Universitari d’Investigació en Atenció Primària Jordi Gol (IDIAP Jordi Gol), 43500 Tortosa, Spain; jlclua.ebre.ics@gencat.cat; 4 Primary Health-Care Center Tortosa Est, Institut Català de la Salut, Primary Care Service (SAP) Terres de l’Ebre, 43500 Tortosa, Spain; 5 Nephrology Department at Dénia Hospital, Conselleria de Sanitat, 03700 Alicante, Spain; 6 Institut d’Educació Secundària El Caminàs, Conselleria d’Educació, 12003 Castellón, Spain; 7 CS Rafalafena, Departament del General de Castelló, Conselleria de Sanitat, 12003 Castellón, Spain; 8 CS Delicias Norte (Zaragoza), Servicio Aragonés de Salud, 50010 Zaragoza, Spain

**Keywords:** major adverse cardiovascular events, atrial fibrillation, chronic kidney disease, machine learning, artificial intelligence

## Abstract

Background: Atrial fibrillation (AF) and chronic kidney disease (CKD) often overlap and may amplify cardiovascular risk. Whether renal dysfunction should be incorporated into composite cardiovascular endpoints in AF remains uncertain. We aimed to quantify AF-associated risk of MACE and evaluate the incremental prognostic value of kidney measures (eGFR and albuminuria) to inform composite outcomes and clinical management. Methods: We performed a retrospective, community-based cohort study of 40,297 adults aged 65–95 years. Individuals with incident AF (*n* = 2574) were followed for 5 years. MACE and components were ascertained from linked health records; only events after AF diagnosis were analyzed. Cox models estimated adjusted hazard ratios (HRs). Risk was further stratified by eGFR stages and urine albumin-to-creatinine ratio (UACR) categories. Exploratory machine learning (ML) was developed to predict MACE in patients with AF and CKD, with model interpretability assessed by feature importance analysis. Results: Incident AF was associated with higher risk of MACE (HR 3.52), CKD (HR 1.97) and all-cause mortality (HR 1.14). CKD was nearly twice more frequent in AF than in non-AF (30.9% vs. 14.5%; *p* < 0.001). Among patients with AF, a graded eGFR–risk relationship was observed: compared with higher eGFR, MACE risk increased across G3a–G5, peaking in G5 (HR 2.08). Albuminuria showed a parallel gradient: versus UACR <30 mg/g, UACR 30–299 mg/g and ≥300 mg/g were associated with an increased risk of MACE (HR 1.51 and 1.76, respectively). Conclusions: Newly diagnosed AF confers a substantial excess risk of MACE and its components. The consistent eGFR and albuminuria in AF support considering clinically meaningful renal endpoints within composite outcomes and prioritizing integrated cardiorenal management. These findings provide actionable evidence to refine risk stratification and endpoint selection in AF research and care.

## 1. Introduction

Atrial fibrillation (AF) is the most frequent sustained arrhythmia in the world [1] and accounts for roughly one-third of hospitalizations for cardiac arrhythmias [2]. It features disorganized atrial activation, resulting in impaired atrial mechanical function. Its estimated prevalence is 1–2% in the general population, rises with age, and the mean age of affected patients is approximately 75 years [3]. AF is associated with a significant increase in major adverse cardiovascular events (MACEs)—including stroke, heart failure, ischemic cardiomyopathy, and mortality—underscoring the importance of early diagnosis and comprehensive management [4,5,6].

Regarding chronic kidney disease (CKD), KDIGO defines CKD as structural or functional abnormalities of the kidney with health implications, present for ≥3 months, and proposes the “C-G-A” classification (cause, G category by GFR, and A category by albuminuria) [7,8]. Estimated glomerular filtration rate (eGFR) is calculated from serum creatinine and/or cystatin C, and albuminuria is assessed using the urine albumin-to-creatinine ratio (UACR) in a spot sample or in 24 h urine. The 2024 KDIGO guidelines recommend screening at-risk individuals using the combination of eGFR and ACR, and confirmation of abnormal findings with repeat testing to establish the diagnosis and stratify prognosis [7,9].

The rising prevalence of CKD—driven by population aging and the expansion of cardiometabolic comorbidities—is independently associated with higher cardiovascular risk, making early detection and control of modifiable factors a priority [10]. Accordingly, there is a bidirectional and clinically relevant relationship between AF and CKD [11]. CKD increases the incidence of AF in a dose-dependent manner, whereas AF is associated with accelerated kidney disease progression and poorer renal outcomes. Recent studies indicate that lower eGFR and higher albuminuria are associated with a greater risk of incident AF [11,12].

Beyond its global burden, CKD is common among individuals with AF, with population-based cohorts reporting substantially higher CKD prevalence in AF than in age-matched controls; this overlap intensifies absolute event rates because CKD clusters with hypertension, diabetes, frailty, and vascular disease [10,11,12,13]. Multiple cohort studies and meta-analyses also show that declining eGFR and UACR track with higher rates of stroke, heart failure, ischemic events, and death, supporting a dose–response relationship between renal impairment and cardiovascular risk in AF [11,12,14]. However, prior literature is often descriptive, heterogeneous in endpoint definitions, and insufficiently multidimensional—frequently evaluating renal or cardiovascular risk in isolation—and composite outcomes seldom incorporate clinically meaningful renal endpoints despite their prognostic weight [11,12,15]. Moreover, widely used AF risk scores (CHADS2, CHA_2_DS_2_-VA or HAS-BLED) do not include renal biomarkers such as eGFR or UACR and may therefore under-estimate risk in patients with CKD, particularly at older ages [4,5,6].

The primary objective of this study was to compare the characteristics of newly diagnosed AF patients to assess the prognostic role of CKD, including renal measures (eGFR and UACR) in the development of MACE.

## 2. Materials and Methods

### 2.1. Study Design

We analyzed the characteristics and nutritional status of 40,297 community-dwelling individuals aged 65–95 years from the general population of the les Terres de l’Ebre region (Catalonia, Spain) who developed AF between 1 January and 31 December 2015, and subsequently experienced MACE from 1 January 2016 through 31 December 2021. This was a retrospective, observational, community-based study including patients with incident AF during a single calendar year and followed for 5 years.

Initially, the cohort comprised residents ≥65 years without a recorded or known history of AF or MACE. A total of 2574 individuals had a new diagnosis of AF and met the inclusion criteria.

This study was conducted in accordance with the Declaration of Helsinki and approved by the Institutional Review Board (or Ethics Committee) of IDIAP Jordi Gol University Institute of Primary Care Research (protocol code, 22/243-P; date of approval, 30 November 2022).

### 2.2. Inclusion and Exclusion Criteria

Inclusion criteria were: (1) age 65–95 years; (2) with no history of AF or prior MACE at baseline; and (3) an active clinical record in any territorial primary care center with data accessible through the Shared Electronic Health Record (HC3) and residence within the region.

Exclusion criteria were: (1) prevalent AF; (2) prevalent MACE; (3) end-stage kidney disease on kidney replacement therapy or kidney transplant at baseline; (4) missing key covariates (age, sex, eGFR, AF status) after imputation checks; (6) implausible laboratory values; and (7) <30 days of potential follow-up after index.

### 2.3. Variables

Data on AF and cardiovascular risk factors were collected until loss to follow-up, death, or 31 December 2021, whichever occurred first. AF was diagnosed according to European Society of Cardiology guidelines. Two physician investigators independently verified the AF diagnosis; disagreements were resolved by consultation with a cardiologist. Patients were classified according to the presence of AF. For AF diagnosed during follow-up, data were captured at the time of AF diagnosis or through the end of follow-up. Only MACEs occurring after the AF diagnosis were analyzed; MACEs preceding AF were not considered. For patients who did not develop AF during follow-up, data were obtained from the final year of observation.

- Sociodemographic variables: age, sex, primary care team, and sub-region.
- Cardiovascular risk factors and diagnoses (ICD-11 code prefixes): hypertension (I10–I15), hypercholesterolemia (E78), body mass index (BMI), diabetes mellitus (E10–E14), sleep apnea–hypopnea syndrome (G47), heart failure (I50–I51), ischemic heart disease (myocardial infarction, percutaneous coronary intervention, stable or unstable angina, or coronary artery bypass grafting) (I20–I25), CKD (N18), UACR (mg/g) and eGFR (mL/min/1.73 m^2^), cerebrovascular disease (transient ischemic attack or ischemic stroke) (G25, I63), chronic obstructive pulmonary disease (J40–J45), cognitive impairment (F06, G31), and cancer (C00–C96).
- Clinical scores: stroke risk by CHA_2_DS_2_-VA and CHA_2_DS_2_-VASc; Barthel Index for basic activities of daily living; Wells score for the probability of deep-vein thrombosis; and the Controlling Nutritional Status (CONUT) score.
- Pharmacologic treatments: antiplatelet agents, direct oral anticoagulants (DOACs), and vitamin K antagonists.
- Vital status at end of follow-up: alive/deceased.

### 2.4. Chronic Kidney Disease Identification and Classification

Prevalent CKD at baseline was defined as either (1) a prior N18 code in the HC3, (2) ≥2 eGFR values < 60 mL/min/1.73 m^2^ separated by ≥90 days, or (3) albuminuria with UACR ≥ 30 mg/g confirmed ≥90 days before the index date, in accordance with KDIGO. Incident CKD during follow-up required fulfillment of KDIGO criteria after the index date with confirmation ≥ 90 days apart [7]. UACR was derived from first-morning spot samples when available; 24 h urine collections were used otherwise.

eGFR was calculated using the CKD-EPI 2021 race-free equation. In sensitivity analyses, we replicated primary models using the CKD-EPI 2009 equation to assess robustness, reporting re-classification across G-stages and the impact on adjusted HRs.

### 2.5. Statistical Analysis

Population characteristics were summarized using standard descriptive statistics. Baseline variables are reported as counts and percentages for categorical data, as means with standard deviations for approximately normally distributed continuous data, and as medians with interquartile ranges otherwise. Categorical variables were compared using the χ^2^ test, and continuous variables with Student’s *t* test for independent samples.

To assess the association between AF and vascular outcomes, hazard ratios (HRs) were estimated using Cox proportional hazards models. HRs were adjusted by including statistically significant confounders in the models. Any variable with a significant *p*-value (*p* ≤ 0.05) that was not already included in the composite scores (CONUT; CHA_2_DS_2_-VA; CHA_2_DS_2_-VASc) was considered a potential confounder. The hazard of MACE was compared between AF and non-AF groups using Cox proportional hazards regression. Statistical analyses and data management were conducted with IBM SPSS Statistics, version 24.0.

Time 0 was the index date (AF diagnosis for the AF cohort; matched index year for non-AF). Participants were censored at the earliest of the following: loss to follow-up, end of observation (31 December 2021), or death. Competing risks. For non-fatal endpoints (stroke, heart failure, ischemic cardiomyopathy and mortality), death was treated as a competing risk; we report cause-specific Cox, HRs, and cumulative incidence.

### 2.6. Machine Learning Development

We developed ML models in individuals with incident atrial fibrillation AF to predict subsequent MACE, using prespecified eligibility criteria. Five algorithms were evaluated: random forest, ExtraTrees, AdaBoost, XGBoost, and LightGBM. Models were trained on the full feature set to predict 1-year risk of MACE and, in a separate task, the occurrence of AF. Development and analysis were conducted in Python 3 with scikit-learn (v1.7) and the XGBoost and LightGBM libraries, selected for their performance and mature tooling.

Feature engineering preceded model training and included systematic screening, selection, and preprocessing of variables. Predictors that were predominantly noisy or highly collinear with stronger features were removed to improve model discrimination and interpretability. Hyperparameters were optimized on the training data via randomized search within cross-validation. Performance was assessed on validation folds using a comprehensive set of metrics—precision, recall, F1-score, and accuracy (and AUROC where applicable)—as detailed in the following section. The best-performing model on validation data was selected a priori as the primary model [6].

To mitigate overfitting, we used cross-validation; systematic hyperparameter optimization; algorithm-specific regularization (e.g., depth constraints, learning-rate shrinkage, and pruning in tree-based models); and feature selection to remove irrelevant or highly collinear predictors. Generalization was then assessed on a held-out test set not used during training or model selection. The dataset was randomly split into a training/development set (two-thirds) and an independent test set (one-third) to enable unbiased evaluation on held-out data.

Shapley additive explanations (SHAPs) were calculated to quantify the contributions of characteristics both globally and for individual predictions. We applied SHAP to the AdaBoost model, as it obtained the best metrics, providing a transparent view of how CKD influences the probability of MACE in patients with AF.

## 3. Results

### 3.1. Baseline Characteristics

Baseline characteristics are summarized in Table 1. We included 2574 individuals with incident AF, of whom 1748 (67.9%) experienced MACE during follow-up. The mean age was 81.22 ± 7.91 years; 53.3% were women, who were significantly older than men (82.23 ± 7.59 vs. 80.53 ± 8.05 years; *p* < 0.001). People with AF had twice the incidence of chronic kidney disease (non-AF 14.5% vs. AF 30.9%, *p* < 0.001).

### 3.2. Association Between AF and Chronic Kidney Disease

In this retrospective, community-based cohort, AF was associated with significantly higher risks across outcomes (Table 2), including a 3.52-fold higher risk of MACE, a 1.97-fold higher risk of CKD, a 4.85-fold higher risk of heart failure, a 2.16-fold higher risk of ischemic heart disease, a 4.03-fold higher risk of stroke, and a 1.14-fold higher risk of all-cause mortality.

### 3.3. Risk of MACE Depending on eGFR

**Figure 1:**
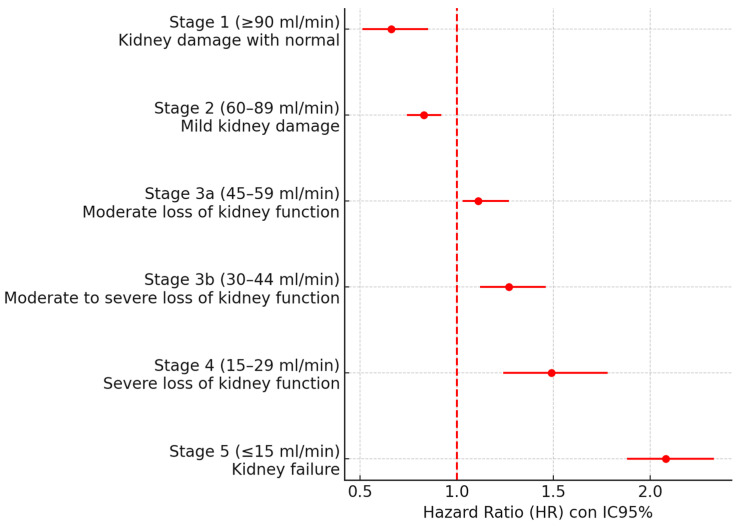
Forest plot according to eGFR.

The risk of MACE was stratified in patients with AF according to their eGFR in Table 3. No significant differences were detected in patients without CKD (stages 1–2), whereas as eGFR decreased, the risk of MACE increased in patients with AF and CKD, with a 2.08-fold higher risk (Figure 1).

### 3.4. Risk of MACE Depending on Albuminuria

The risk of MACE was stratified in patients with AF according to their AUCR in Table 4. Normal to moderate albuminuria was associated with a 15% increase in the risk of MACE HR 1.51 (1.47–1.55), and normal to severe albuminuria was associated with a 26.4% increase in HR 1.76 (1.67–1.81).

## 4. Discussion

New-onset AF was associated with a substantial increase in the risk of MACE (HR 3.52), as well as higher risks of heart failure (HR 4.85), stroke/TIA (HR 4.03), ischemic heart disease (HR 2.16), and all-cause mortality (HR 1.14). In addition, CKD was nearly twice as frequent among individuals who developed AF (30.9% vs. 14.5%; *p* < 0.001), and renal dysfunction displayed a consistent risk gradient. Among patients in G3a–G5, the risk of MACE increased progressively, peaking in G5 (HR 2.08). Complementing eGFR, albuminuria showed a parallel, stepwise association with outcomes in our cohort: compared with normal UACR (<30 mg/g), MACE proportions rose from 60.0% to 75.0% and 86.4% in the moderate (30–299 mg/g) and severe (≥300 mg/g) categories, respectively, with corresponding HRs of 1.51 (95% CI 1.47–1.55) and 1.76 (1.67–1.81), while lower risk was observed in the normal range (HR 0.78, 0.69–0.88). Taken together, these findings underscore the centrality of the cardio–renal axis in the natural history of AF and suggest that incorporating clinically meaningful renal endpoints into composite outcomes may better capture overall disease burden than conventional MACE definitions focused exclusively on cardiovascular events.

The bidirectional AF–CKD association reported in population-based studies and meta-analyses was corroborated in our cohort: lower eGFR and higher albuminuria are associated with AF, and conversely, AF is linked to more rapid kidney disease progression and worse renal and cardiovascular outcomes [7,14,15,16]. Our observation of increased MACE—and of each component—after incident AF, together with the stepwise rise in risk as eGFR declines, is consistent with large cohorts [15] and prior reviews positioning CKD as a risk amplifier for stroke, heart failure, coronary ischemia, and death in AF [15,17,18,19,20]. Albuminuria, meanwhile, involves hemodynamic load, inflammation and altered glomerular permeability; its presence and magnitude are associated with endothelial dysfunction, vascular stiffness and myocardial remodeling, which fits with the observed gradient of MACE risk by UACR categories. From a mechanistic standpoint, chronic inflammation, RAAS activation, atrial remodeling due to volume overload, and shared comorbidities (age, hypertension, diabetes, obesity) provide plausible pathways underlying the observed gradient [18,19,21].

A practical implication of our work is that the risk attributable to renal dysfunction reaches magnitudes comparable to classic MACE components in AF. This supports the hypothesis that CKD—operationalized by clinically meaningful sustained eGFR decline, progression to advanced G stages, or initiation of kidney replacement therapy—should be considered for inclusion in AF composite endpoints, particularly in older populations where AF–CKD coexistence is common [7,14,17]. Similarly, our UACR data support the inclusion of renal endpoints based on albuminuria in event composites, and the combination of eGFR and UACR in prognostic stratification in HF, both to quantify risk and to select cardiorenal interventions. In parallel, KDIGO 2024 emphasizes combined screening with eGFR and albuminuria and confirmation of abnormal results to refine diagnosis and risk stratification, reinforcing the clinical relevance of integrating the renal dimension into AF risk assessment [7]. This supports the implementation of care pathways in AF that include periodic measurement of UACR, action thresholds, and targeted follow-up in A ≥ 2 subgroups, where the potential clinical benefit appears to be greater.

These cohort results align with external evidence from a recent systematic review and meta-analysis [14], showing that any albuminuria versus none increases incident AF risk (RR 1.43, 95% CI 1.25–1.63), and that moderately to severely increased albuminuria (UACR ≥ 30 mg/g) versus <30 mg/g confers higher risk (RR 1.64, 1.31–2.06), with a graded association across UACR categories. This meta-analytic pattern mirrors our dose–response findings and supports the role of albuminuria as an independent, incremental marker beyond eGFR for AF-related risk.

Our results further indicate that patients with AF and CKD require intensive, integrated management strategies, aligned with contemporary guidelines: optimal control of blood pressure and glycemia, albuminuria reduction, use of cardiorenal-protective therapies where indicated, and anticoagulation tailored to thromboembolic risk and renal function [7,18,19,22]. eGFR-based stratification identifies AF subgroups at particularly high risk (G3b–G5), in whom the potential clinical benefit of close follow-up, cardiorenal therapy optimization, and targeted interventions addressing frailty/nutrition may be greater [23]. Given the prognostic role of UACR, therapeutic prioritization should consider drugs with proven antiproteinuric and cardiorenal effects, as well as albuminuria reduction targets as measures of response to treatment.

Moreover, our findings support including renal endpoints within MACE composites in AF research and practice. Beyond enhancing the clinical validity of the composite, this approach may increase sensitivity for detecting the benefit of interventions that act on both the cardiovascular and renal axes.

Although we evaluated ML models for predicting MACE after AF using multiple algorithms, a unifying result emerged: the diagnosis of CKD and renal variables (eGFR and albuminuria) ranked among the most informative predictors, in keeping with recent reports comparing ML with traditional clinical scores [4,6]. This suggests that renal markers not only improve risk discrimination but also enhance clinical interpretability.

Recent advances in AI across cardiology—spanning cardiac imaging interpretation, ECG-based arrhythmia detection from ambulatory and wearable devices, heart-failure phenomapping, and multimodal risk stratification—demonstrate that data-driven models can deliver clinically meaningful improvements when they are interpretable, well-calibrated, and evaluated for clinical utility [24]. In line with this experience, our machine learning analyses consistently ranked kidney variables (eGFR and albuminuria) among the top predictors of MACE in AF, reinforcing the biological centrality of the cardio-renal axis and supporting cardiorenal-aware models for risk stratification. From a translational perspective, explainability helps align model behavior with mechanistic plausibility, while calibration metrics and decision-curve analysis clarify whether incremental discrimination translates into better decision making at realistic treatment thresholds. To responsibly advance toward implementation, external validation across health-system settings, fairness analyses across age/sex and renal strata, prospective impact evaluation, and post-deployment monitoring for temporal drift will be essential. Collectively, these considerations position eGFR and albuminuria as high-value, interpretable features for AI-enabled prognosis in AF, and provide a rationale to test composite endpoints that explicitly integrate clinically meaningful renal outcomes.

This study leverages a large, community-based sample of incident AF with 5-year follow-up, physician verification of AF diagnoses, and estimation of incidence rates and adjusted HRs. eGFR stratification provides a biologically coherent gradient with direct clinical relevance. Nevertheless, the retrospective observational design entails potential residual confounding and misclassification. In particular, heterogeneous availability and possible underreporting of UACR may induce differential/non-differential classification bias and attenuate true associations; furthermore, point measurement of UACR may not capture intra-individual variability. External validation is needed to generalize these findings to other settings. In the ML component, despite validation and regularization procedures, models may still be susceptible to overfitting, temporal drift, and may require recalibration before deployment in different environments.

## 5. Conclusions

Newly diagnosed atrial fibrillation was associated with a notable increase in major adverse cardiovascular events and its components. With the support of machine learning analysis, renal dysfunction showed an independent and clinically relevant risk gradient. The inclusion of chronic kidney disease, estimated glomerular filtration rate measurement, and urine albumin-to-creatinine ratio should be considered as endpoints in atrial fibrillation, as should conventional major adverse cardiovascular events components, and integrated cardiorenal treatment should be prioritized. These actions will implement renal information in atrial fibrillation care and provide justification for renal assessment in trials and clinical practice.

## Figures and Tables

**Table 1:** Baseline characteristics of cases with AF vs. without AF.

Variables	No AF	(%)	AF	(%)	*p*	ALL
All (*n* %)	37,723	93.6%	2574	6.4%	-	40,297
Women	17,535	46.5%	1343	3.3%	<0.001	18,878
Age average	77.6 ± 8.7		81.2 ± 7.9		<0.001	77.9 ± 8.5
CHA_2_DS_2_-VASc	3.2 ± 1.2		3.8 ± 1.2		<0.001	3.2 ± 1.2
CHA_2_DS_2_-VA	2.6 ± 1.1		3.4 ± 1.2		<0.001	2.7 ± 1.1
Hypertension arterial	23,610	62.6%	1945	75.6%	<0.001	25,555
Diabetes mellitus	9689	25.7%	769	30%	<0.001	10,458
Dyslipidemia	17,913	47.5%	1216	47.3%	0.822	19,129
BMI ^1^ (kg/m^2^)	28.7 ± 5.1		29.5 ± 5.4		<0.001	28.7 ± 5.2
Ischemic cardiomyopathy	2558	6.8%	357	13.9%	<0.001	2915
Heart failure	2096	5.6%	676	26.·%	<0.001	2772
Stroke/TIA	698	1.9%	187	7.3%	<0.001	885
Vascular peripheral disease	2431	6.4%	345	13.4%	<0.001	2776
Dementia/cognitive impairment	3471	9.2%	310	12.1%	<0.001	3781
Chronic kidney disease	5481	14.5%	795	30.9%	<0.001	5834
Glomerular filtration rate (mL/min/1.73 m^2^)	72.9 ± 18.6		63.5 ± 20.4		<0.001	72.2 ± 19
OSAHS ^2^	1022	2.7%	126	4.9%	<0.001	1148
COPD ^3^/asthma/bronchitis	4591	12.2%	447	17.4%	<0.001	5038
Statins	11,806	31.3%	945	36.7%	<0.001	12,751
Antiaggregants	6110	16.2%	141	5.5%	<0.001	6251
Anticoagulation	987	2.6%	1994	77.5%	<0.001	2981
VKA ^4^	754	2%	944	36.7%	<0.001	1698
NOAC ^5^	235	0.6%	1053	40.9%	<0.001	1288
CHARLSON	1.3 ± 1.3		1.8 ± 1.4		<0.001	1.3 ± 1.3
Average follow-up time	80.8 ± 9.3		78.6 ± 12.1		<0.001	80.7 ± 9.5

^1^ OSAHS: sleep apnea–hypopnea syndrome;^2^ COPD: chronic obstructive pulmonary disease; ^3^ BMI: body mass index; ^4^ VKA: vitamin K antagonist; ^5^ NOAC: non-vitamin K antagonist oral anticoagulant.

**Table 2:** Association between AF diagnosis and MACE.

	No-AF	New AF	HR AF/No-AF
N	37,723	2574	
AF (*n*) Incidence/1000 people-years [CI95%]	-	2574 8.9 [8.6–9.2]	-
Chronic kidney disease (n %) Incidence/1000 people-years [CI95%]	5481 (14.52%) 20.3 [19.8–20.9]	795 (30.88%) 40.1 [37.1–43.2]	1.97 [1.82–2.13] *p* < 0.001
Heart failure (n %) Incidence/1000 people-years [CI95%]	2096 (5.56%) 8.3 [7.9–8.6]	676 (26.26%) 40.1 [37.1–43.2]	4.85 [4.5–5.3] *p* < 0.001
Ischemic heart disease (n %) Incidence/1000 people-years [CI95%]	2558 (6.78%) 10.1 [9.7–10.5]	367 (14.26%) 21.8 [19.6–24.1]	2.16 [1.93–2.41] *p* < 0.001
Stroke/transient ischemic attack (n %) Incidence/1000 people-years [CI95%]	698 (1.85%) 2.7 [2.5–3.0]	187 (7.26%) 11.1 [9.6–12.8]	4.03 [3.43–4.74] *p* < 0.001
Death (n %) Incidence/1000 people-years [CI95%]	6799 (18.02%) 26.8 [26.1–27.4]	518 (20.12%) 30.7 [28.1–33.5]	1.14 [1.04–1.25] *p* = 0.027
Total MACE (n%) Incidence/1000 people-years [CI95%]	5352 (14.11%) 21.1 [20.5–21.6]	1748 (67.9%) 73.0 [68.9–77.1]	3.52 [3.31–3.75] *p* < 0.001

**Table 3:** Stratification of MACE risk depending on eGFR in patients with AF.

Stage		Estimated Glomerular Filtration Rate (eGFR)	AF + MACE+	AF + MACE-	HR IC95%	Total AF
1		eGFR ≥ 90 mL/min (Kidney damage with normal)	42	106	0.66 (0.51–0.85)	148
2		eGFR 60–89 mL/min (Mild kidney damage)	371	604	0.83 (0.74–0.92)	975
3	a	eGFR 45–59 mL/min (Moderate loss of kidney function)	175	208	1.11 (1.03–1.27)	383
	b	eGFR 30–44 mL/min (moderate to severe loss of kidney function)	121	112	1.27 (1.12–1.46)	133
4		eGFR 15–29 mL/min (Severe loss of kidney function)	52	33	1.49 (1.24–1.78)	85
5		eGFR ≤ 15 mL/min (Kidney failure)	53	64	2.08 (1.88–2.33)	117

**Table 4:** Stratification of MACE risk depending on albuminuria in patients with AF.

Albuminuria	AF + MACE+	AF + MACE−	HR IC95%	Total AF
<30 mg/g (normal)	850	566	0.78 (0.69–0.88)	1416
30–299 mg/g (moderate)	675	225	1.51 (1.47–1.55)	901
≥300 mg/g (severe)	222	35	1.76 (1.67–1.81)	257

## Data Availability

The data presented in this study are available on request from the corresponding author due to the privacy reasons (sensitive data).
